# Combining Three Peripheral Blood Biomarkers to Stratify Rheumatoid Arthritis–Associated Interstitial Lung Disease Risk

**DOI:** 10.1002/acr.70008

**Published:** 2026-02-24

**Authors:** Kelsey Coziahr, Austin M. Wheeler, Brent A. Luedders, Michael Duryee, Halie Frideres, Katherine D. Wysham, Grant W. Cannon, Gary Kunkel, Dana P. Ascherman, Paul A. Monach, Gail S. Kerr, Andreas M. Reimold, Scott M. Matson, Joshua F. Baker, Geoffrey M. Thiele, Ted R. Mikuls, Bryant R. England

**Affiliations:** ^1^ Veterans Affairs (VA) Nebraska‐Western Iowa Health Care System Omaha; ^2^ University of Nebraska Medical Center Omaha; ^3^ VA Puget Sound Health Care System Seattle Washington; ^4^ University of Washington Seattle; ^5^ Salt Lake City VA Health Care System Salt Lake City Utah; ^6^ University of Utah Salt Lake City; ^7^ Pittsburgh VA Health Care System Pittsburgh Pennsylvania; ^8^ University of Pittsburgh Pittsburgh Pennsylvania; ^9^ VA Boston Health Care Boston Massachusetts; ^10^ Washington DC VA Medical Center Washington DC; ^11^ Georgetown University Washington DC; ^12^ Howard University Washington DC; ^13^ Dallas VA Medical Center Dallas Texas; ^14^ University of Texas Southwestern Dallas; ^15^ Kansas University Medical Center Kansas City; ^16^ Corporal Michael J. Crescenz VA Medical Center Philadelphia Pennsylvania; ^17^ University of Pennsylvania Philadelphia

## Abstract

**Objective:**

The purpose was to evaluate a biomarker score consisting of *MUC5B* rs35705950 promoter variant, plasma matrix metalloproteinase‐7 (MMP‐7), and serum anti–malondialdehyde‐acetaldehyde (anti‐MAA) antibody for rheumatoid arthritis (RA)–associated interstitial lung disease (ILD) risk stratification.

**Methods:**

Using a multicenter cohort of US veterans with RA, we performed a cross‐sectional study of prevalent RA‐ILD and a cohort study of incident RA‐ILD. Medical records were used to confirm clinical ILD diagnoses by chest imaging or lung biopsy pathology reports. A combined three‐biomarker score (range 0–3) was calculated based on the presence of the *MUC5B* variant and elevations (upper 25% vs lower 75%) in MMP‐7 and anti‐MAA antibody concentrations. Multivariate logistic and Cox regression models were adjusted for clinical risk factors.

**Results:**

Among 2,043 participants with RA, prevalent ILD was identified in 88 (88.7% male; mean age 63.6 years). The odds of prevalent RA‐ILD were higher with increased biomarker score (adjusted odds ratio 12.21 [95% confidence interval (CI) 3.82–38.97] for score of 3 vs 0). A score ≥1 had 76.1% sensitivity but 48.6% specificity. Incident RA‐ILD developed in 148 participants, with those having a combined biomarker score of 3 having the highest risk (adjusted hazard ratio 4.36 [95% CI 1.55–12.27]). The area under the curve for prevalent RA‐ILD and Harrell's C for incident RA‐ILD were highest when clinical risk factors were combined with the biomarker score.

**Conclusion:**

This three‐analyte biomarker score was associated with both prevalent and incident RA‐ILD, improving risk stratification beyond clinical risk factors. Although this score is inadequate for clinical implementation, these findings demonstrate the potential for biomarker scores in RA‐ILD risk stratification.

## INTRODUCTION

Rheumatoid arthritis (RA) affects approximately 0.5% to 1% of the US population.^1^ Pulmonary manifestations have long been recognized to occur in RA, with much emphasis focused on interstitial lung disease (ILD) given its poor prognosis.^2^ Despite RA treatment advances, ILD remains the most overrepresented cause of death in RA.^3^ The estimated prevalence of ILD in RA varies widely across studies, typically ranging from 10% to 40% of patients with RA.^4^

**SIGNIFICANCE & INNOVATIONS**

- A simple biomarker score composed of *MUC5B*, matrix metalloproteinase‐7, and anti–malondialdehyde‐acetaldehyde antibody was significantly associated with rheumatoid arthritis–associated interstitial lung disease (RA‐ILD) risk.
- Adding the three‐biomarker score to clinical risk factors significantly improved RA‐ILD risk stratification beyond clinical factors alone.
- Given the narrow scoring range of 0 to 3, no cut point produced suitable sensitivity and specificity adequate for clinical implementation.
- These findings show the potential for uncomplicated multibiomarker scores to enable RA‐ILD risk stratification and screening. RA‐ILD is typically identified through advanced imaging studies, specifically high‐resolution computed tomography (HRCT) of the chest, after patients have developed respiratory‐related symptoms such as cough or shortness of breath. There is no standard screening protocol for selecting which patients to refer for chest CT, or when to refer them, before respiratory symptoms develop. Thus, there is a concern that RA‐ILD is not detected early in the disease course when it may be more amenable to treatment. Recent American College of Rheumatology (ACR)/American College of Chest Physicians (CHEST) clinical practice guidelines emphasize the role of HRCT and pulmonary function tests (PFTs) for evaluating for RA‐ILD among those at higher risk.^5^ However, only a few clinical risk factors were summarized, leaving some uncertainty in whom to screen.

In addition to providing valuable evidence on disease pathogenesis, studies evaluating peripheral biomarkers have also highlighted their potential role for enhancing risk stratification and ultimately facilitating the earlier identification and treatment of RA‐ILD.^6^ The *MUC5B* rs35705950 promoter variant, the strongest genetic risk factor for idiopathic pulmonary fibrosis, has been shown to carry significant risk of RA‐ILD, specifically the usual interstitial pneumonia (UIP) pattern of ILD.^7^ *MUC5B* status meaningfully influences lifetime risk of developing ILD among people with RA, with those possessing risk alleles having a 17% risk compared with only 6% for those without risk alleles.^8^ Beyond genetics, plasma matrix metalloproteinase‐7 (MMP‐7) concentrations have been found to strongly improve discrimination of prevalent RA‐ILD,^9^, ^10^, ^11^ as well as stratify the risk for incident RA‐ILD.^11^ Although routinely obtained RA autoantibodies, rheumatoid factor and anti–cyclic citrullinated peptide (anti‐CCP) antibody, have limited value in RA‐ILD prediction, serum anti–malondialdehyde‐acetaldehyde (anti‐MAA) antibody concentrations are independently associated with prevalent and incident RA‐ILD risk.^12^, ^13^, ^14^ Lung tissues from patients with RA‐ILD have also demonstrated enhanced staining for MAA antigen.^12^ Recognizing each of these biomarkers has been implicated in RA‐ILD pathogenesis, it remains to be determined how different pathways (eg, autoimmunity, inflammation, fibrosis, extracellular matrix remodeling, and mucociliary dysfunction) may act to drive RA‐ILD onset.^12^, ^15^, ^16^ Moreover, with these biomarkers individually demonstrating strong associations with RA‐ILD, the question remains if combining these peripheral biomarkers could improve RA‐ILD risk stratification.

The purpose of this study was to test a simplified combined biomarker scoring system using the *MUC5B* promoter variant, plasma MMP‐7, and serum anti‐MAA antibody to risk stratify prevalent and incident ILD in a large multicenter prospective RA cohort. We hypothesized that this three‐biomarker score would improve risk stratification compared with clinical risk factors alone.

## PATIENTS AND METHODS

### Study design and population

We completed a cross‐sectional study of prevalent RA‐ILD and a cohort study of incident RA‐ILD using the Veterans Affairs Rheumatoid Arthritis (VARA) registry. The VARA registry is a multicenter, prospective cohort of US veterans with rheumatologist‐diagnosed RA that was initiated in 2003.^17^ All participants fulfill the 1987 ACR classification criteria for RA.^18^ Institutional review board (IRB) approval is maintained at all sites, and all participants provided written informed consent. This study was approved by the Veterans Affairs (VA) Nebraska‐Western Iowa Health Care System and VA Central IRBs.

Participants were required to have replete biomarker data to be eligible for analyses. In cross‐sectional analyses, we excluded participants with indeterminate RA‐ILD and those who developed RA‐ILD after registry enrollment (ie, incident RA‐ILD). In cohort analyses, we excluded indeterminate RA‐ILD and RA‐ILD diagnosed before registry enrollment (ie, prevalent RA‐ILD).

### Demographics and clinical data

Patient demographics were self‐reported upon enrollment in the VARA registry, including date of birth, sex, race and ethnicity, and cigarette smoking history. Race and ethnicity was self‐identified as either American Indian/Pacific Islander, Asian American, Black and African American, Hispanic, or White. Cigarette smoking history was classified as either current, former, or never smoking.

Additional clinical data used in analyses included body mass index (BMI) category, anti‐CCP positivity, Disease Activity Score in 28 joints (DAS28), and Rheumatic Disease Comorbidity Index (RDCI) score. BMI was calculated from the most recent weight and modal height in the health record at registry enrollment. Anti‐CCP was measured on banked serum samples collected at enrollment using a second‐generation enzyme‐linked immunosorbent assay (ELISA). ACR core RA disease activity measures (eg, joint counts, global assessments, and acute phase reactants) were collected longitudinally as part of routine care by the treating providers to score the DAS28.^19^, ^20^ For these analyses, we used the enrollment DAS28 score. The RDCI score at enrollment was used as a measure of comorbidity burden,^21^ and components were identified by diagnostic codes from linked administrative data. Medication use including methotrexate, biologic/targeted‐synthetic disease‐modifying antirheumatic drugs (b/tsDMARDs), prednisone, and conventional synthetic DMARDs were recorded at the time of registry enrollment.

### RA‐ILD assessment

RA‐ILD was assessed in the VARA registry through a validated two‐stage process.^22^, ^23^ Firstly, the entire cohort is screened for ILD diagnostic codes from medical encounters and CT reports with ILD‐related terms. Subsequently, potential RA‐ILD cases undergo systematic medical record review to validate RA‐ILD diagnoses. RA‐ILD is considered validated based on the treating providers’ diagnosis of ILD and supportive findings of ILD from chest imaging (eg, chest CT) or lung biopsy pathology reports. ILD was considered indeterminate when confirmatory clinical diagnoses and supportive imaging or biopsy findings were not present. Chest CT was not universally performed in all participants. RA‐ILD pattern was extracted from the medical records, when available. We also considered chest CT reports with honeycombing to be evidence of UIP. Forced vital capacity (FVC) percentage predicted was extracted from the medical records. Participants with RA‐ILD and other pulmonary manifestations (eg, chronic obstructive pulmonary disease and bronchiectasis) were included as having RA‐ILD.

### Combined biomarker score

A combined biomarker score (range 0–3) was calculated from the presence of the *MUC5B* rs35705950 variant and elevations in MMP‐7 and anti–MAA‐albumin antibody concentrations. Each of the three biomarkers have been studied individually but not combined into a scoring system.^7^, ^8^, ^11^, ^12^, ^13^, ^24^ The *MUC5B* variant was measured by the Infinium Global Screening Array 2.0 (Illumina), and autosomal dominant inheritance was assumed for the T variant allele. MMP‐7 was measured from banked plasma using the Meso Scale Discovery platform (Meso Scale Diagnostics). Serum anti‐MAA antibodies (IgM isotype) were measured using an in‐house ELISA. Elevations in MMP‐7 and anti‐MAA antibody concentrations were defined as high versus normal corresponding to the upper 25% and the lower 75% of continuous values, as in prior work.^11^, ^12^ A combined biomarker score (range 0–3) was calculated based on the presence of the *MUC5B* variant (1 point) and elevations (upper 25% vs lower 75%) in MMP‐7 and anti‐MAA antibody concentrations (1 point for each).

### Statistical analysis

Patient characteristics were descriptively summarized. Cross‐sectional analyses of the three‐biomarker score with prevalent RA‐ILD were completed using multivariable logistic regression models adjusting for age, sex, race, smoking status, BMI category, anti‐CCP antibody positivity, DAS28, and RDCI score. These covariates were selected based on their potential to confound the relationship between biomarker and RA‐ILD and, notably, included all risk factors for RA‐ILD in the ACR/CHEST.^5^ Receiver operating characteristic (ROC) curves were generated and the area under the ROC curve (AUC) was calculated, comparing models with clinical variables alone versus models with clinical variables and the combined biomarker score. Sensitivity, specificity, negative predictive value, and positive predictive value were calculated for prevalent RA‐ILD based on the combined biomarker scores. In sensitivity analyses, these metrics were calculated for each combination of two of the three evaluated biomarkers. In secondary analyses, the associations of the biomarker score with UIP RA‐ILD and non‐UIP RA‐ILD were tested in separate models.

In incident RA‐ILD analyses, participants were observed from registry enrollment until RA‐ILD diagnosis, death, or end of study period (June 30, 2021), and median time to ILD diagnosis was calculated. Incidence rates and 95% confidence intervals (CIs) were estimated using the Stata strate command. Multivariable Cox regression models were used to assess the combined biomarker score and incident RA‐ILD risk, adjusting for the covariates described previously. Harrell's C was calculated to compare models with clinical variables alone versus models with clinical variables and the combined biomarker score. Missing continuous covariables were imputed using the average value from multiple imputation with chained equations with 10 imputations. All analyses were completed using Stata version 18 (StataCorp LLC) within the VA Informatics and Computing Infrastructure.

## RESULTS

### Prevalent RA‐ILD analyses

#### Patient characteristics

Among 2,043 participants with RA, prevalent ILD was identified in 88. Participants were predominantly male (88.7%), most commonly self‐reported as White (77.4%), and had a mean age of 63.6 (SD 11.2%) years (Table 1). The *MUC5B* promotor variant was present in 17.5% of those with prevalent ILD. Combined biomarker scores of 0 (47.6%) and 1 (40.7%) were more common than scores of 2 (10.6%) or 3 (1.1%). Participants with higher combined scores tended to be older, had higher disease activity scores, and were more likely to be anti‐CCP antibody positive and current smokers. Methotrexate and b/tsDMARD use were less common among those with high combined biomarker scores. Among those with prevalent RA‐ILD, the mean FVC percentage predicted was 76.9 (SD 18.6), 77.3 (SD 16.5), 71.0 (SD 15.6), and 67.3 (SD 19.4) for biomarker scores of 0, 1, 2, and 3, respectively.

**Table 1:** Participant characteristics for prevalent RA‐ILD analyses by combined biomarker score*

	Overall (n = 2,043)	Score = 0 (n = 972)	Score = 1 (n = 832)	Score = 2 (n = 217)	Score = 3 (n = 22)
Age, mean (SD) y	63.6 (11.2)	61.6 (11.7)	64.8 (10.3)	67.0 (11.2)	70.0 (7.3)
Male, %	88.7	86.9	90.3	91.7	81.8
White, %	77.4	75.9	77.3	83.9	86.4
Smoking status, %					
Current	24.7	23.3	26.6	22.1	40.9
Former	52.9	50.3	54.8	58.5	40.9
Never	20.4	23.4	17.5	18.4	18.2
Missing	2.0	3.1	1.1	0.9	0.0
BMI, %					
<25	24.6	23.9	24.9	25.8	36.4
≥25 to <30	39.9	40.7	39.8	37.3	36.4
≥30 to <35	23.8	24.1	22.8	26.3	22.7
≥35	11.6	11.3	12.5	10.6	4.5
RDCI score, mean (SD)	3.31 (1.91)	3.16 (1.96)	3.39 (1.86)	3.68 (1.84)	3.63 (1.26)
Anti‐CCP positive, %	76.6	74.5	76.6	84.3	95.5
DAS28, mean (SD)	3.76 (1.50)	3.66 (1.48)	3.77 (1.49)	4.16 (1.53)	4.14 (1.25)
MDHAQ, mean (SD)	0.88 (0.62)	0.84 (0.61)	0.90 (0.64)	0.96 (0.60)	0.73 (0.57)
Methotrexate, %	49.2	52.3	47.7	42.9	36.4
b/tsDMARDs, %	26.3	25.9	26.8	26.7	18.2
Prednisone, %	18.2	18.8	17.2	17.5	36.4
csDMARDs, %	73.3	75.2	73.1	66.4	68.2

* Analytes included in score were *MUC5B* promoter variant, plasma matrix metalloproteinase‐7, and serum anti–malondialdehyde‐acetaldehyde antibody. BMI, body mass index; b/tsDMARD, biologic/targeted‐synthetic disease‐modifying antirheumatic drug; CCP, cyclic‐citrullinated peptide; csDMARD, conventional synthetic disease‐modifying antirheumatic drug; DAS28, Disease Activity Score in 28 joints; MDHAQ, Multidimensional Health Assessment Questionnaire; RA‐ILD, rheumatoid arthritis–associated interstitial lung disease; RDCI, Rheumatic Disease Comorbidity Index.

#### Biomarker performance

Among participants with prevalent RA‐ILD, biomarker scores of 2 (25.0%) and 3 (5.7%) were more common than among participants without ILD (10.0% and 0.9%, respectively; Table 2). In multivariable models, higher combined biomarker scores were associated with greater odds of prevalent RA‐ILD. Odds of prevalent RA‐ILD were >2‐fold higher for a score of 1 (adjusted odds ratio [aOR] 2.14 [95% CI 1.24–3.72]), >4‐fold higher for a score of 2 (aOR 4.25 [95% CI 2.21–8.14]), and >12‐fold higher for a score of 3 (aOR 12.21 [95% CI 3.82–38.97]) relative to a score of 0. Test of trend across scores was highly significant (*P* < 0.001). When assessing UIP and non‐UIP RA‐ILD separately, associations of the biomarker score were stronger for UIP, although 95% CIs were overlapping (Supplemental Table 1).

**Table 2:** *MUC5B*, MMP‐7, and anti‐MAA antibody combined biomarker score and odds of prevalent RA‐ILD*

Combined biomarker score	RA without ILD (n = 1,955), n (%)	RA‐ILD (n = 88), n (%)	aOR (95% CI)
0	951 (48.6)	21 (23.8)	1 (ref)
1	792 (40.5)	40 (45.5)	2.14 (1.24, 3.72)
2	195 (10.0)	22 (25.0)	4.25 (2.21, 8.14)
3	17 (0.9)	5 (5.7)	12.21 (3.82, 38.97)

* Models adjusted for age, sex, race, smoking status, body mass index category, anti–cyclic citrullinated peptide antibody positivity, Disease Activity Score in 28 joints, and Rheumatic Disease Comorbidity Index score. aOR, adjusted odds ratio; CI, confidence interval; MAA, malondialdehyde‐acetaldehyde; MMP, matrix metalloproteinase; RA‐ILD, rheumatoid arthritis–associated interstitial lung disease.

For all RA‐ILD, the sensitivity of a combined biomarker score ≥1 was 76.1% but dropped substantially when required to be ≥2 (30.7%) or 3 (5.7%; Supplemental Table 2). Specificity was poor with a score of ≥1 (48.6%) but increased to 89.2% and 99.1% for cutoffs of ≥2 and ≥3, respectively. Positive predictive values were low and negative predictive values were high at all cutoffs. Sensitivity was reduced without corresponding gains in specificity when limiting the combined three‐biomarker score to only two of the biomarkers (ie, *MUC5B* and MMP‐7 only; Supplemental Table 3).

**Figure 1:**
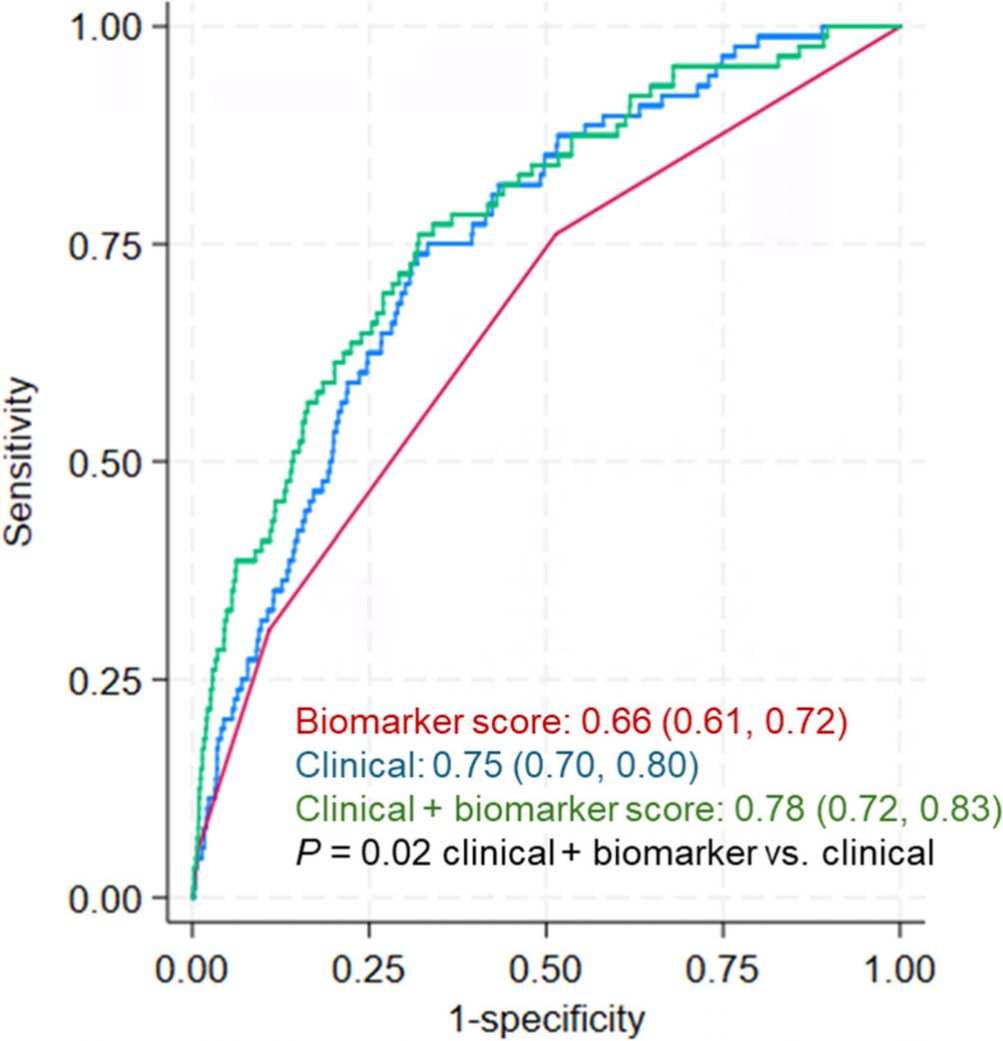
Receiver operating characteristic curve comparing clinic factors and a combined biomarker score for RA‐ILD. Receiver operating characteristic curve combined biomarker score only, clinical factors only, and clinical plus biomarker models for discriminating prevalent RA‐ILD. Values are area under the curve and 95% confidence intervals. Biomarkers included the *MUC5B* promoter variant, matrix metalloproteinase‐7, and anti–malondialdehyde‐acetaldehyde adduct antibodies. Clinical risk factors included age, sex, race, smoking status, body mass index category, anti‐CCP antibody positivity, Disease Activity Score in 28 joints, and Rheumatic Disease Comorbidity Index score. CCP, cyclic citrullinated peptide; RA‐ILD, rheumatoid arthritis–associated interstitial lung disease.

The combined biomarker score alone discriminated RA‐ILD with an AUC of 0.66 (95% CI 0.61–0.72; Figure 1). When adding the combined biomarker score to clinical factors, the AUC improved from 0.75 (95% CI 0.70–0.80) to 0.78 (95% CI 0.72–0.83; *P* = 0.02 for difference).

### Incident RA‐ILD analyses

#### Patient characteristics

Participants in the cohort study were without prevalent ILD and had similar characteristics as those in the prevalent analyses (Table 3). Again, participants with higher combined biomarker scores were older, had higher disease activity scores, and were more likely to be anti‐CCP antibody positive and current smokers.

**Table 3:** Participant characteristics for incident RA‐ILD analyses by combined biomarker score*

	Overall (n = 2,103)	Score = 0 (n = 999)	Score = 1 (n = 858)	Score = 2 (n = 225)	Score = 3 (n = 21)
Age, mean (SD), y	63.4 (11.1)	61.6 (11.6)	64.7 (10.2)	66.4 (10.8)	68.1 (9.2)
Male, %	88.8	87.3	90.0	91.6	81.0
White, %	76.9	75.8	77.2	80.4	85.7
Smoking status, %					
Current	25.1	23.3	27.2	23.1	42.9
Former	52.4	50.8	54.2	54.7	38.1
Never	20.7	23.2	17.7	21.3	19.0
Missing	1.8	2.7	0.9	0.9	0.0
BMI, %					
<25	24.3	23.8	24.4	26.2	28.6
≥25 to <30	40.4	41.0	40.0	39.1	42.9
≥30 to <35	23.7	24.2	23.0	24.0	23.8
≥35	11.6	10.9	12.7	10.7	4.8
RDCI score, mean (SD)	3.27 (1.89)	3.14 (1.93)	3.36 (1.88)	3.47 (1.76)	3.57 (1.33)
Anti‐CCP positive, %	76.7	74.5	76.1	82.2	95.2
DAS28, mean (SD)	3.77 (1.50)	3.68 (1.50)	3.77 (1.48)	4.16 (1.55)	4.09 (1.16)
MDHAQ, mean (SD)	0.88 (0.63)	0.84 (0.61)	0.90 (0.64)	0.95 (0.61)	0.88 (0.62)
Methotrexate, %	51.1	53.5	49.9	45.8	47.6
b/tsDMARDs, %	26.0	25.4	26.8	26.2	19.0
Prednisone, %	18.3	19.2	16.9	18.7	23.8
csDMARDs, %	74.1	75.9	73.9	67.8	61.9

* Analytes included in score were *MUC5B* promoter variant, plasma matrix metalloproteinase‐7, and serum anti–malondialdehyde‐acetaldehyde antibody. BMI, body mass index; b/tsDMARD, biologic/targeted‐synthetic disease‐modifying antirheumatic drug; CCP, cyclic citrullinated peptide; csDMARD, conventional synthetic disease‐modifying antirheumatic drug; DAS28, Disease Activity Score in 28 joints; MDHAQ, Multidimensional Health Assessment Questionnaire; RA‐ILD, rheumatoid arthritis–associated interstitial lung disease; RDCI, Rheumatic Disease Comorbidity Index.

#### Biomarker score performance

**Figure 2:**
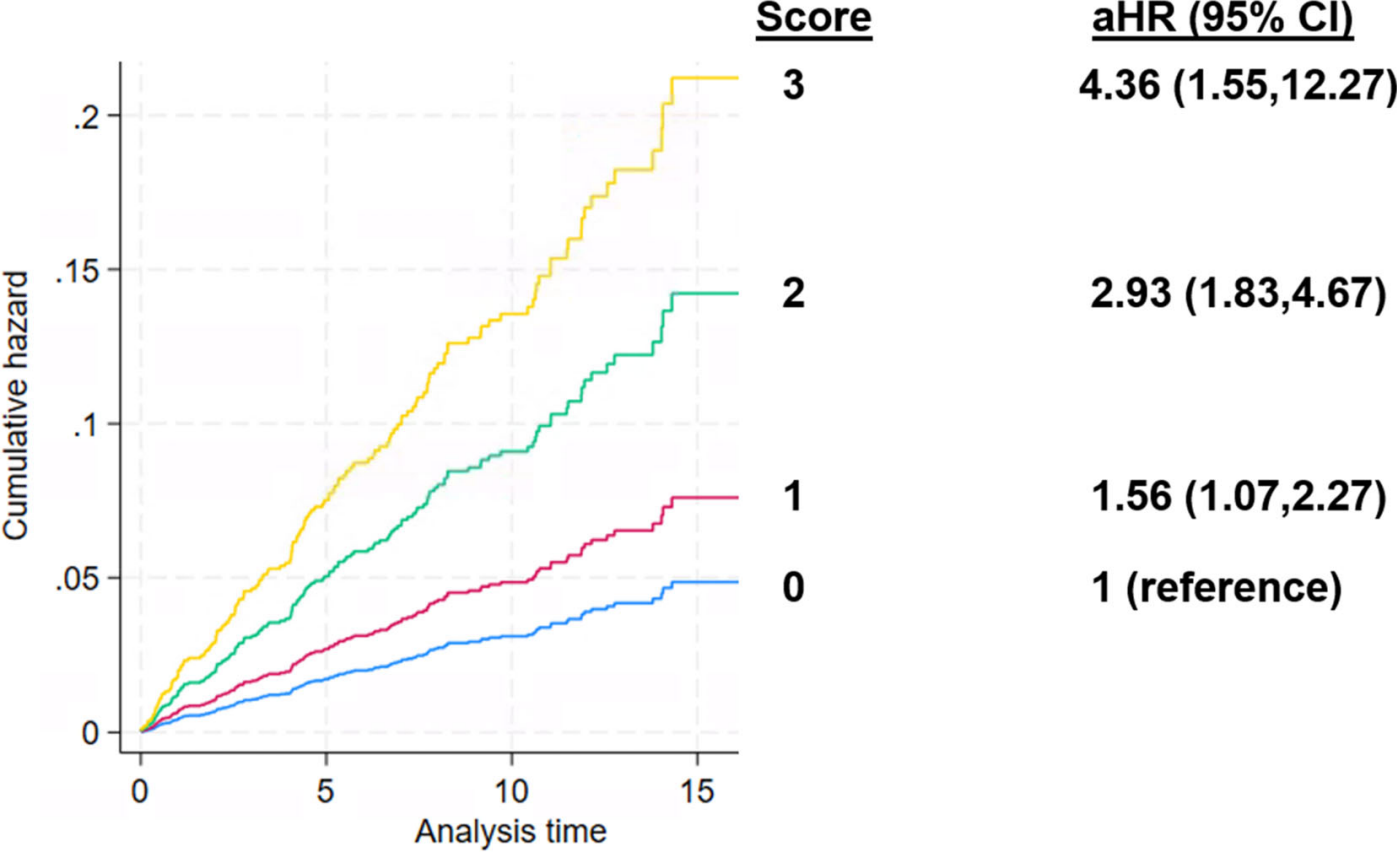
*MUC5B*, MMP‐7, and anti‐MAA antibody combined biomarker score and incident RA‐ILD risk. Cumulative hazard of incident RA‐ILD by a combined biomarker score composed of the *MUC5B* promoter variant, MMP‐7, and anti‐MAA adduct antibodies. Models were adjusted for age, sex, race, smoking status, body mass index category, anti‐CCP antibody positivity, Disease Activity Score in 28 joints, and Rheumatic Disease Comorbidity Index score. aHR, adjusted hazard ratio; CCP, cyclic citrullinated peptide; CI, confidence interval; MAA, malondialdehyde‐acetaldehyde; MMP, matrix metalloproteinase; RA‐ILD, rheumatoid arthritis–associated interstitial lung disease.

Incident RA‐ILD developed in 148 of 2,103 participants over 17,748 patient‐years (PY) of follow‐up. The median time to RA‐ILD diagnosis was 5.1 years for a score of 0, 4.0 years for a score of 1, 2.6 years for a score of 2, and 3.7 years for a score of 3. RA‐ILD incidence increased from 187.5 (95% CI 141.3–248.7) per 1,000 PY for a combined biomarker score of 0 to 274.2 (95% CI 102.9–730.6) for a combined biomarker score of 3 (Table 4). Higher combined biomarker scores were associated with a significantly increased risk of incident ILD. Relative to those with a score of 0, the adjusted hazard ratio was 1.56 (95% CI 1.07–2.27) for a score of 1, 2.93 (95% CI 1.83–4.67) for a score of 2, and 4.36 (95% CI 1.55–12.27) for a score of 3 (Table 4; Figure 2). The test of trend across the score was highly significant (*P* < 0.001). Combining the biomarker score with clinical factors best predicted RA‐ILD incidence with a Harrell's C index of 0.68 compared with 0.62 for the biomarker score alone (*P* = 0.001) and 0.65 for clinical findings alone (*P* = 0.05).

**Table 4:** RA‐ILD incidence rate by combined biomarker score*

Score	n	n incident ILD/PY follow‐up	IR per 1,000 PY (95% CI)	Unadjusted HR (95% CI)	Adjusted HR (95% CI)^a^
0	999	48/8,831.0	187.5 (141.3–248.7)	1 (reference)	1 (reference)
1	858	66/7,145.2	212.8 (167.2–270.9)	1.70 (1.17–2.46)	1.56 (1.07–2.27)
2	225	30/1,635.2	221.7 (155.0–317.0)	3.33 (2.11–5.26)	2.93 (1.83–4.67)
3	21	4/136.5	274.2 (102.9–730.6)	5.21 (1.88–14.45)	4.36 (1.55–12.27)

* CI, confidence interval; HR, hazard ratio; ILD, interstitial lung disease; IR, incidence rate; PY, person years; RA‐ILD, rheumatoid arthritis–associated interstitial lung disease.

^a^ Adjusted for age, sex, race, smoking status, body mass index category, anti–cyclic citrullinated peptide positivity, Disease Activity Score in 28 joints, and Rheumatic Disease Comorbidity Index score.

## DISCUSSION

Recognizing the absence of effective risk stratification and screening strategies currently available for RA‐ILD, we aimed to test whether a simple biomarker score based on three peripheral blood biomarkers previously associated with RA‐ILD individually could improve risk stratification of prevalent and incident RA‐ILD beyond clinical risk factors alone. We found that a simple biomarker score derived from the *MUC5B* rs35705950 promoter variant, plasma MMP‐7, and serum anti‐MAA antibody was associated with a higher risk of prevalent and incident RA‐ILD in a large multicenter prospective cohort of US veterans with RA. Over half of the RA cohort had a score of ≥1, with the odds of prevalent RA‐ILD being 2‐ to 12‐fold higher and incident ILD risk being 1.5‐ to >4‐fold higher among this group. In contrast, only 2% of patients with RA with a score of 0 had prevalent RA‐ILD. Thus, a simple three‐biomarker score based on *MUC5B*, MMP‐7, and anti‐MAA antibody shows potential for risk stratifying prevalent and incident RA‐ILD and, upon further refinement and validation in other cohorts, could inform RA‐ILD screening.

There is growing interest in identifying peripheral blood biomarkers that could aid in RA‐ILD risk stratification, with more than 90 peripheral blood biomarkers having been found to be associated with RA‐ILD in a recent systematic literature review.^2^ How these biomarkers could be effectively used alone or in combination has yet to be determined. Some prior studies have used broad biomarker profiling approaches, such as machine learning, to discriminate the presence or absence of RA‐ILD.^6^, ^25^, ^26^ Although this approach retains the most information from large‐scale biomarker profiling, it may overfit models to the derivation sample data and is unlikely to be practical in clinical settings. Alternatively, high‐performing biomarkers could be selectively combined into more parsimonious panels.^10^ This can be of particular benefit when trying to boost the sensitivity of the biomarker panel and/or limit costs associated with measuring a large number of analytes. In this study, we used the latter approach, combining three promising biomarkers into a simple biomarker score. Despite its simplicity, this score was independently and strongly associated with RA‐ILD risk. Notably, this finding was not limited to prevalent RA‐ILD, and this study is among the first to show that a peripheral blood biomarker score is associated with incident RA‐ILD risk.

To guide RA‐ILD screening, an effective risk stratification tool should possess excellent sensitivity and sufficient specificity, enabling the selection of high‐risk individuals to undergo guideline‐recommended testing with HRCT and PFTs.^5^ For prevalent RA‐ILD, a biomarker score of 1 or greater had 76% sensitivity, but the specificity was only 48%. Raising this threshold to 2 or greater markedly improved the specificity to 89%, but the sensitivity correspondingly dropped to 30%. Thus, this score can possess sufficient sensitivity or specificity but not both at the same score cutoff. The sensitivity and specificity were not improved with any combination of only two biomarkers (Supplemental Table 3). Our intent for simplicity in the combined biomarker score likely contributed to these large jumps in performance metrics across the narrow score range. Additionally, because no clinically relevant thresholds have been established, we categorized high and low values for MMP‐7 and anti‐MAA based on quartiles. Thus, our findings represent a first step in establishing a simple multibiomarker score for RA‐ILD. Combining this biomarker score with emerging RA‐ILD clinical risk scores^27^ and exploring alternative cut points for MMP‐7 and anti‐MAA concentrations in future work could address current performance limitations. Although modification of the biomarker score examined herein may boost performance, this relatively simple biomarker score composed of only three analytes improved risk stratification of prevalent and incident RA‐ILD beyond clinical risk factors in its current form.

There were limitations to the study. The study population was composed of primarily White men which may limit generalizability. However, RA‐ILD has a predilection to affect men.^28^ Participants with higher biomarker scores were systematically different from those with lower biomarker scores. Although we adjusted for these differences, there may be residual or unmeasured confounding variables. Cigarette smoking history was collected as current, former, or never at the time of registry enrollment. We were unable to account for smoking intensity or duration as well as changes in smoking status that may have occurred during follow‐up. ILD pattern was not available for all participants, which limited the power of our ILD subgroup analyses. Participants with RA‐ILD may have had concomitant alternative pulmonary diseases such as chronic obstructive pulmonary disease or bronchiectasis which could influence findings. The three biomarkers investigated in this study have previously been associated with RA‐ILD individually within this cohort.^11^, ^12^, ^24^ Although each biomarker has been implicated in RA‐ILD pathogenesis, the exact pathophysiology linking these distinct pathways (eg, autoimmunity, inflammation, fibrosis, extracellular matrix remodeling, and mucociliary dysfunction) has yet to be determined.^12^, ^15^, ^16^ However, others have also observed strong associations between *MUC5B* and MMP‐7 with RA‐ILD.^7^, ^8^, ^9^, ^13^ Although the biomarker score statistically improved RA‐ILD risk stratification beyond clinical risk factors, the performance was modest. External validation of this biomarker score is needed. Finally, the cost‐effectiveness of using a combined three‐biomarker score as part of RA‐ILD screening remains to be determined.

In conclusion, we established that a simple biomarker score based on *MUC5B*, MMP‐7, and anti‐MAA antibody was independently associated with prevalent and incident RA‐ILD. Our findings demonstrate how select panels of peripheral biomarkers may be able to enable RA‐ILD screening by identifying a high‐risk group who should undergo chest CT imaging and completion of PFTs. Further studies identifying environmental risk factors and other informative peripheral biomarkers and combining these into effective yet practical risk scores are needed to enable earlier detection and intervention in RA‐ILD.

## AUTHOR CONTRIBUTIONS

All authors contributed to at least one of the following manuscript preparation roles: conceptualization AND/OR methodology, software, investigation, formal analysis, data curation, visualization, and validation AND drafting or reviewing/editing the final draft. As corresponding author, Dr England confirms that all authors have provided the final approval of the version to be published and takes responsibility for the affirmations regarding article submission (eg, not under consideration by another journal), the integrity of the data presented, and the statements regarding compliance with institutional review board/Declaration of Helsinki requirements.

## Supporting information


**Disclosure Form**:

**Supplemental Table 1** Association of combined biomarker score with RA‐ILD by ILD pattern.
**Supplemental Table 2**. Performance of Combined Biomarker Score at Different Cutoffs for Prevalent RA‐ILD.
**Supplemental Table 3**. Performance of Combined Biomarker Score at Different Cutoffs for Prevalent RA‐ILD using only two biomarkers.
