# Supplementary material for: Combining Three Peripheral Blood Biomarkers to Stratify Rheumatoid Arthritis–Associated Interstitial Lung Disease Risk
Source: Arthritis Care Res (Hoboken). 2026 Feb 24;78(8):1016–23. doi: 10.1002/acr.70008 (PMC13420941; doi:10.1002/acr.70008)
Supplement: Supplementary file 2 — Supplemental Table 1 Association of combined biomarker score with RA‐ILD by ILD pattern. Supplemental Table 2. Performance of Combined Biomarker Score at Different Cutoffs for Prevalent RA‐ILD. Supplemental Table 3. Performance of Combined Biomarker Score at Different Cutoffs for Prevalent RA‐ILD using only two biomarkers. [file ACR-78-1016-s002.docx]

**SUPPLEMENTARY MATERIALS**

Combining Three Peripheral Biomarkers to Stratify Rheumatoid Arthritis-Associated Interstitial Lung Disease Risk

Coziahr et al.

**Supplemental Table 1**. Association of combined biomarker score with RA-ILD by ILD pattern.

**Supplemental Table 2**. Performance of Combined Biomarker Score at Different Cutoffs for Prevalent RA-ILD.

**Supplemental Table 3**. Performance of Combined Biomarker Score at Different Cutoffs for Prevalent RA-ILD using only two biomarkers.

| **Supplemental Table 1.**Association of combined biomarker score with RA-ILD by ILD pattern. | | |
| --- | --- | --- |
| **Score** | **UIP aOR (95% CI)** | **Non-UIP aOR (95% CI)** |
| 0 | Ref | Ref |
| 1 | 3.73 (1.66, 8.34) | 1.20 (0.54, 2.66) |
| 2 | 6.43 (2.54, 16.28) | 3.09 (1.24, 7.65) |
| 3 | 25.33 (6.20, 103.51) | 4.96 (0.56, 44.02) |
| *adjusted for age, sex, race, smoking status, BMI category, anti-CCP positivity, DAS28, and Rheumatic Disease Comorbidity Index Score  N=52 UIP RA-ILD; n=36 no-UIP RA-ILD | | |

**Supplemental Table 2**. Performance of Combined Biomarker Score at Different Cutoffs for Prevalent RA-ILD.

| Score | Sensitivity (%) | Specificity (%) | NPV (%) | PPV (%) |
| --- | --- | --- | --- | --- |
| ≥1 | 76.1 | 48.6 | 97.8 | 6.3 |
| ≥2 | 30.7 | 89.2 | 96.6 | 11.3 |
| 3 | 5.7 | 99.1 | 95.9 | 22.7 |

Abbreviations: NPV, negative predictive value; PPV, positive predictive value.

**Supplemental Table 3**. Performance of Combined Biomarker Score at Different Cutoffs for Prevalent RA-ILD using only two biomarkers.

|  | Sensitivity (%) | Specificity (%) | PPV (%) | NPV (%) |
| --- | --- | --- | --- | --- |
| *MUC5B* & anti-MAA     ≥1     2 | 53.4  12.5 | 64.0  96.5 | 6.3  13.8 | 96.8  96.1 |
| *MUC5B* & MMP-7     ≥1     2 | 67.1  18.2 | 63.8  96.1 | 7.7  17.2 | 97.7  96.3 |
| Anti-MAA & MMP-7     ≥1     2 | 62.5  11.4 | 58.6  94.9 | 6.4  9.1 | 97.2  96.0 |

Abbreviations: anti-MAA, anti-malondialdehyde acetaldehyde antibodies; MMP-7, matrix metalloproteinase-7; NPV, negative predictive value; PPV, positive predictive value.
